# Large Orbital Magnetic Moment in VI_3_

**DOI:** 10.1021/acs.nanolett.2c04045

**Published:** 2023-02-01

**Authors:** Dávid Hovančík, Jiří Pospíšil, Karel Carva, Vladimír Sechovský, Cinthia Piamonteze

**Affiliations:** † Department of Condensed Matter Physics, Faculty of Mathematics and Physics, Charles University, Ke Karlovu 5, 121 16Prague 2, Czech Republic; ‡ Swiss Light Source, Paul Scherrer Institut, CH-5232Villigen PSI, Switzerland

**Keywords:** 2D van der Waals magnet, X-ray magnetic circular dichroism, orbital moment, VI_3_

## Abstract

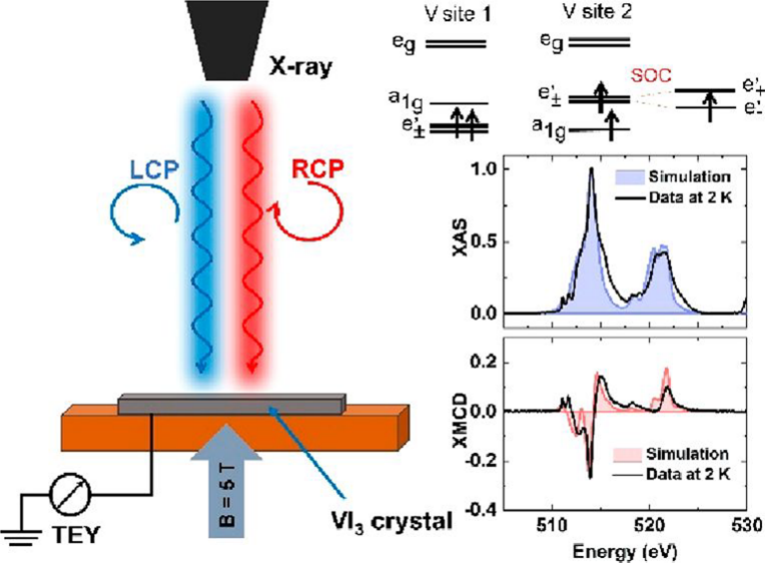

The existence of the V^3+^-ion orbital moment is an open issue of the nature of magnetism in the van der Waals ferromagnet VI_3_. The huge magnetocrystalline anisotropy in conjunction with the significantly reduced ordered magnetic moment compared to the spin-only value provides strong but indirect evidence of a large V orbital moment. We used the unique capability of X-ray magnetic circular dichroism to determine the orbital component of the total magnetic moment and provide a direct proof of an exceptionally sizable orbital moment of the V^3+^ ion in VI_3_. Our ligand field multiplet simulations of the XMCD spectra in synergy with the results of DFT calculations agree with the existence of two V sites with different orbital occupations and OM magnitudes in the ground state.

Two-dimensional (2D) van der Waals (vdW) magnetic materials have recently attracted ever-increasing interest from materials researchers because their stable magnetic ordering, even in atomically thin monolayers, provides great opportunities for spintronic devices.^1−5^ The magnetic ordering in 2D relies on magnetic anisotropy since Mermin and Wagner^6^ have shown that long-range magnetic order cannot exist in the one- or two-dimensional isotropic Heisenberg model. As proposed by Bruno,^7^ the magnetocrystalline anisotropy originates from anisotropy in the orbital moment (OM). In the extensively studied CrI_3_, the electronic configuration of the Cr^3+^ ion which experiences an octahedral crystal field is *S* = 3/2, *L* = 0. The small magnetic OM value below 0.1 μ_B_ was confirmed by XMCD measurements.^8^ As proposed by Kim et al.,^9^ the magnetic anisotropy of CrI_3_ comes from the iodine p orbital spin–orbit coupling (SOC) and the p–d covalence between Cr and I, hence, being relatively small. Here we focus on a Mott insulator VI_3_, which began to be intensively investigated after the papers of Son et al.,^10^ Kong et al.,^11^ and Tian et al.^12^ on ferromagnetism in bulk crystals were published. The recent work of Lin et al.^13^ showing an anomalous increase of *T*_C_ for one or few monolayers of VI_3_ further boosted this interest. In VI_3_ the electrons in a partially filled  level may possess an effective OM of magnitude given by the quantum number *l* = 1,^14^ which for the occupancy of two electrons leads to *S* = 1, *L* = 1 configuration. As *S* and *L* are antiparallel due to the less than half-filled V d-shell, one would expect a total magnetic moment of 1 μ_B_, if the picture is not affected by other effects quenching the orbital moment. Early publications on VI_3_^10−12^ showed huge disagreement on the magnitude of the out-of-plane (*c*^R^-axis; *c*^R^ stands for *c*-axis in rhombohedral structure) magnetic moment (at 2 K and 5 T the values 2.5, 1.25, and 1.1 μ_B_/V, respectively). Later, Liu et al. reported a moment of 1 μ_B_/V and proposed an unquenched OM.^15^ This scenario was further supported by neutron diffraction experiment done by Hao et al.,^16^ which found a magnetic moment of 1.2 μ_B_/V at 6 K. Electronic structure calculations have found a ground state where the  orbital is half occupied leading to an unquenched OM.^17,18^ Strong correlations represented by Hubbard *U* are sufficient to open a gap between SO split bands so that this solution is semiconducting. On the other hand, another calculation suggests different semiconducting ground state with fully occupied  orbitals,^19^ which leads to a negligible orbital moment. Direct evidence and quantification of an unquenched OM is still lacking.

TX_3_ transition metal trihalides (T = transition metal, X = Cl, Br, I) adopt two common layered crystal structures with T^3+^ ions arranged in a honeycomb network at the edge-sharing octahedral coordination by six X ions (see Figure 2a). VI_3_ undergoes a structural transition at 79 K between the high-temperature rhombohedral  and low-temperature monoclinic  variant.^11,20−23^ Below 50 K it orders ferromagnetically with an easy-magnetization direction tilted by ∼40° from the *c*^R^-axis.^16,24^ A strong magnetic anisotropy is observed, reinforcing the proposition of an unquenched OM,^25^ which was recently found to give rise to strong anisotropy also in FePS_3_.^26^ Around 36 K a second magnetic phase transition was proposed separating the states with one (at temperatures between 36 and 50 K) and two inequivalent magnetically ordered V sites (at lower temperatures).^27^ In addition, a symmetry lowering from monoclinic to a triclinic structure appears at 32 K upon cooling.^23,28^ Magnetotransport measurements also show a qualitatively different ferromagnetic state below 40 K, compared to the one between *T*_c_ and 40 K.^29^

Motivated by the multiple hints for nonzero OM in VI_3_, we utilized the unique ability of X-ray magnetic circular dichroism (XMCD) to determine spin and orbital moments separately^30,31^ in an element-specific fashion. Our results confirm the existence of an exceptionally large OM. Ligand field multiplet simulations of the XAS and XMCD spectra are in qualitative agreement with the existence of two V sites with different orbital occupations and therefore different OM at 2 K.

Magnetization measurements show a 1.23 μ_B_/V-ion along the easy magnetization direction, well matching the neutron-experiment result^16^ (see Supporting Information, Figure S1). Angular dependence of 5 T magnetization in the *ac*^R^-plane at 2 K shows that the easy axis is tilted ∼40° from the *c*^R^-axis, also in agreement with previous publications.^16,24,32^

Figure 1a shows the V L_3,2_ (2p_3/2,1/2_ → 3d, between 505 and 530 eV) helicity-summed XAS of VI_3_ and XMCD spectra derived as σ^+^(ω) – σ^–^(ω) measured at 2 K and 5 T applied parallel to the *c*^R^-axis. The XAS spectrum agrees well with the V^3+^ valence state published for V_2_O_3_ and the previous XAS published for VI_3_.^33,34^ For the 2 K spectrum besides the V L_3,2_ absorption edges, we detected another two resonances above 530 eV which were identified as the O K absorption edge;^35^ see Figure 1b. The 90 K XAS measurement (see Supporting Information, Figure S2) shows an oxygen-free spectrum (Figure 1b shows XAS spectrum taken at 300 K as reference) measured after the low-temperature data, clearly showing that the O_2_ is not intrinsic to the sample surface. The likely origin of the oxygen contribution is discused in the Supporting Information.

**Figure 1:**
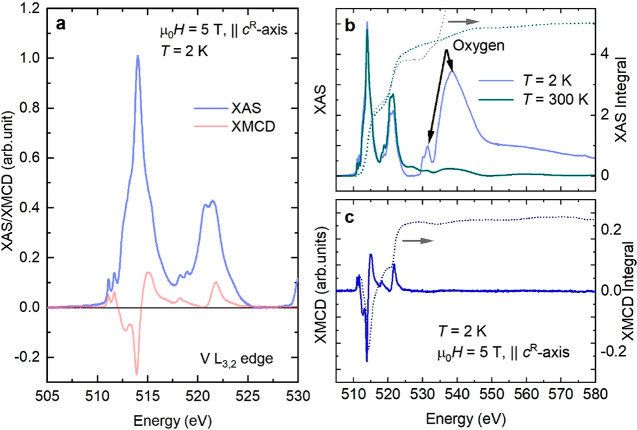
(a) V L_3,2_ XAS spectra of VI_3_ after removing the step function and XMCD spectrum measured at 2 K. (b) XAS spectra in extended energy region and corresponding XAS integrals (dotted lines). (c) XMCD spectrum at 2 K (continuous line) and corresponding integral (dotted line).

From the XMCD integral shown in Figure 1c, one can notice that the value of the integrated intensity is finite and positive. According to the magneto-optical sum rules of the XMCD spectra^30,31^ (see Supporting Information, eq S1) the total integral of the XMCD signal is proportional to the OM. Therefore, the positive finite integral undoubtedly confirms the nonzero out-of-plane (*c*^R^-axis) OM component antiparallel to the spin moment, as expected. Applying the magneto-optical orbital sum rules to the spectra (for  where  is the number of holes in the 3d orbital), we obtain *m*_orb_ = 0.6 (1) μ_B_. The error bar takes into account the uncertainty in determining the XAS integral due to the oxygen contribution, as detailed in the Supporting Information. The spin sum rule cannot be applied to V spectra due to the large overlap of states from the L_3_ and L_2_ edges, which makes the calculation of a correction factor unfeasible.^36,37^

In VI_3_ a trigonal distortion leads to a splitting of  level into  singlet and  doublet. Two possible ground states for 3d^2^ configurations are (i) lower energy of  doublet, which would lead to the ground state with a fully occupied  level and orbital singlet with *L* = 0; (ii) electronic occupation of  levels where the  doublet is further split by spin–orbit interaction (see Figure 2b,c,d). In the second case, the electron in the  level can be assigned an effective moment analogous to p electrons with *l* = 1, and the ground state may be in the high OM state with *L*= 1.

**Figure 2:**
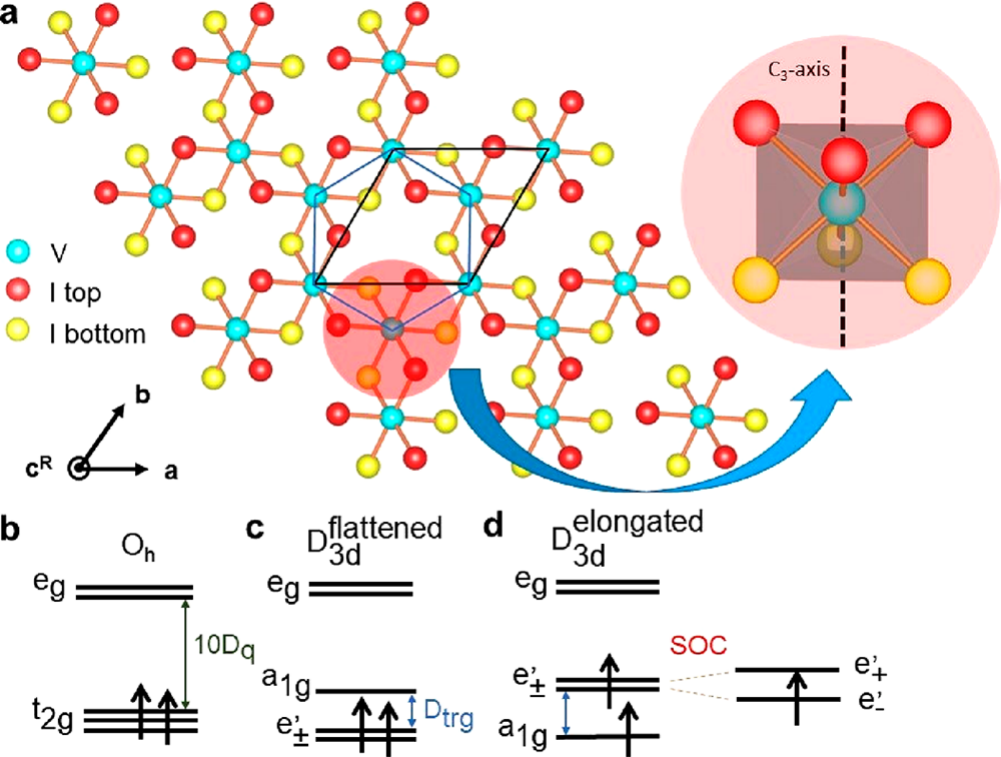
(a) Crystal structure of VI_3_ monolayer. The VI_6_ cluster is emphasized (the 3-fold axis C_3_ is along the *c*^R^-axis). (b–d) Crystal field splitting for *O_h_* and *D*_3*d*_ (flattened/elongated octahedra) symmetry and corresponding electron occupation.

In the case of a trigonally distorted cubic lattice, one can decide which of the two above cases is energetically favorable knowing whether the cubic body diagonal (along C_3_-axis) is elongated or shortened. In VI_3_ the symmetry of more distant neighbors of V beyond the octahedral I cage differs significantly from the octahedral one (see Figure 2), which may lead to corrections (of trigonal symmetry) comparable to that of the small I cage distortion. Therefore, we have employed the electronic structure DFT (for details see Supporting Information, DFT) calculations to get a more accurate picture. When both the spin–orbit interaction and correlation effects in terms of Hubbard *U* are included (we used *U* = 4.3 eV and Hund’s exchange *J* = 0.8 eV), these calculations converge to two strikingly different solutions: either a state with quenched OM, typical for 3d transition metals, or a state with an exceptionally high OM.^17,18^ The latter state is energetically favorable, but the energy difference is small, approximately 5.6 meV according to our calculation. Under some circumstances, the state with quenched OM may become preferred; see Supporting Information, Note S1. For the spin moment, all calculations predict values close to 2 μ_B_ in agreement with Hund rules.

For the solution with high OM, the orbital-resolved DOS for the spin-up (Figure 3 a) shows that the  states are almost fully occupied while  states are empty. Notably the relevant bands are rather broad and  character bands have a different evolution in *k*-space than  bands. Nevertheless, one can say that  states are generally energetically lower than  as expected for this situation. On the other hand, the solution with the quenched OM (Figure 3 b) shows for the spin-up a complete occupation of  and  states whereas the  orbital states are above the Fermi level. This solution was found previously using the VASP code with a smaller Hubbard *U* = 2 eV.^19^

**Figure 3:**
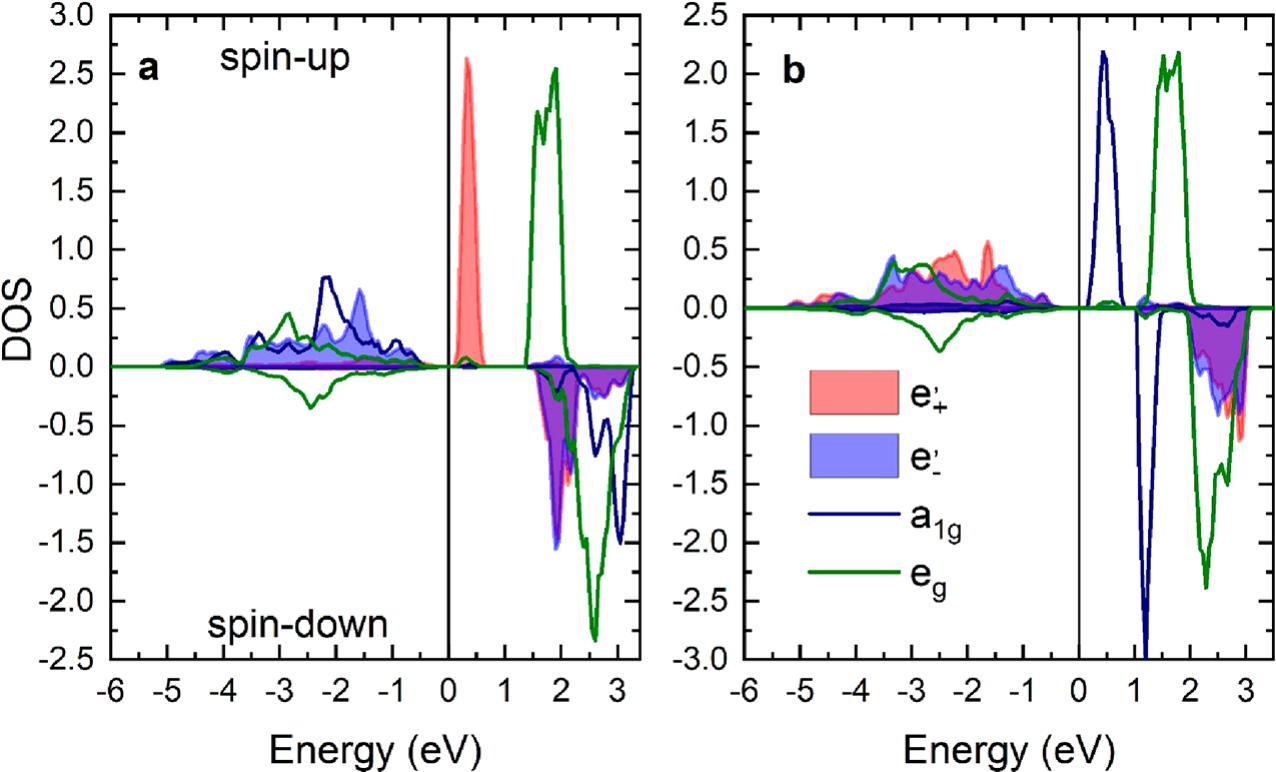
Calculated orbital-resolved DOS of VI_3_ for spin-up/down V 3d electrons: (a) solution with high 3d OM value; (b) solutions with quenched OM. Note the violet area is the overlap of the  (in blue) and  (in red) states.

To help understanding of the V ground state, we have also performed ligand multiplet simulation of the XAS and XMCD spectra incorporating the strong interaction between the 2p core hole and 3d electrons.^38^ Given the broadness of some of the bands, the use of an *ab initio* electronic structure to obtain parameters for the multiplet calculation has to be done with caution. From the calculation performed without Hubbard *U* in order to look at single electron energy levels we estimate  splitting  to be in the range of 1.3–1.8 eV. For the multiplet simulation, we have used  = 1.5 eV from this range. Other parameters and details of the simulation are described in the Supporting Information.

Figure 4 shows the measured spectra compared to simulations with evaluated projections of spin and orbital angular momentum into the field direction,  and  We define quantities  and  as angular momentum divided by the reduced Planck constant ℏ. Blue curves (Figure 4a,b) correspond to a negative  (for definition see eq S2 in Supporting Information) energy splitting which leads to  orbital as the lowest in energy. This simulation corresponds to  for V in the ground state at 10 K. The red simulation (Figure 4b,c), on the other hand, corresponds to a positive  of the  levels with  as the lowest energy orbital giving  The largest discrepancy for the simulation with  is an additional positive peak at the L_2_ XMCD around 520 eV. The  ground state simulation shows a different intensity ratio compared to the data for the positive and negative peaks at the L_3_-edge XMCD between 513 and 515 eV. The  simulation gives an unquenched OM, in qualitative agreement with the experimental finding; however, the size of *m*_orb_ is overestimated by a factor of 2, approximately. In the inelastic neutron scattering experiments, Lane et al.^39^ have found that their observations fit only with a system composed of two simultaneously coexisting V sites with opposite trigonal distortion, where one leads to the quenched OM while the other one leads to an unquenched OM. Various kinds of coexisting different stable states for V have already been suggested in other works.^17,27,40^ The *d* SOC and crystal field parameters used for the simulations shown in Figure 4 are in close agreement with those used by Lane et al. To simulate the coexistence of two V sites with opposite distortions, we take the average (50% occupancy each) of the simulations for  and  ground state which is represented by the green curve in Figure 4e,f. The qualitative agreement between data and simulation is improved in comparison to the single site simulations. Most importantly, the average OM from the two sites, which corresponds to 0.59 μ_B_ is in very good agreement with the value obtained from the XMCD orbital sum rule. Therefore, our results support the picture of two coexisting inequivalent V magnetic sites.

**Figure 4:**
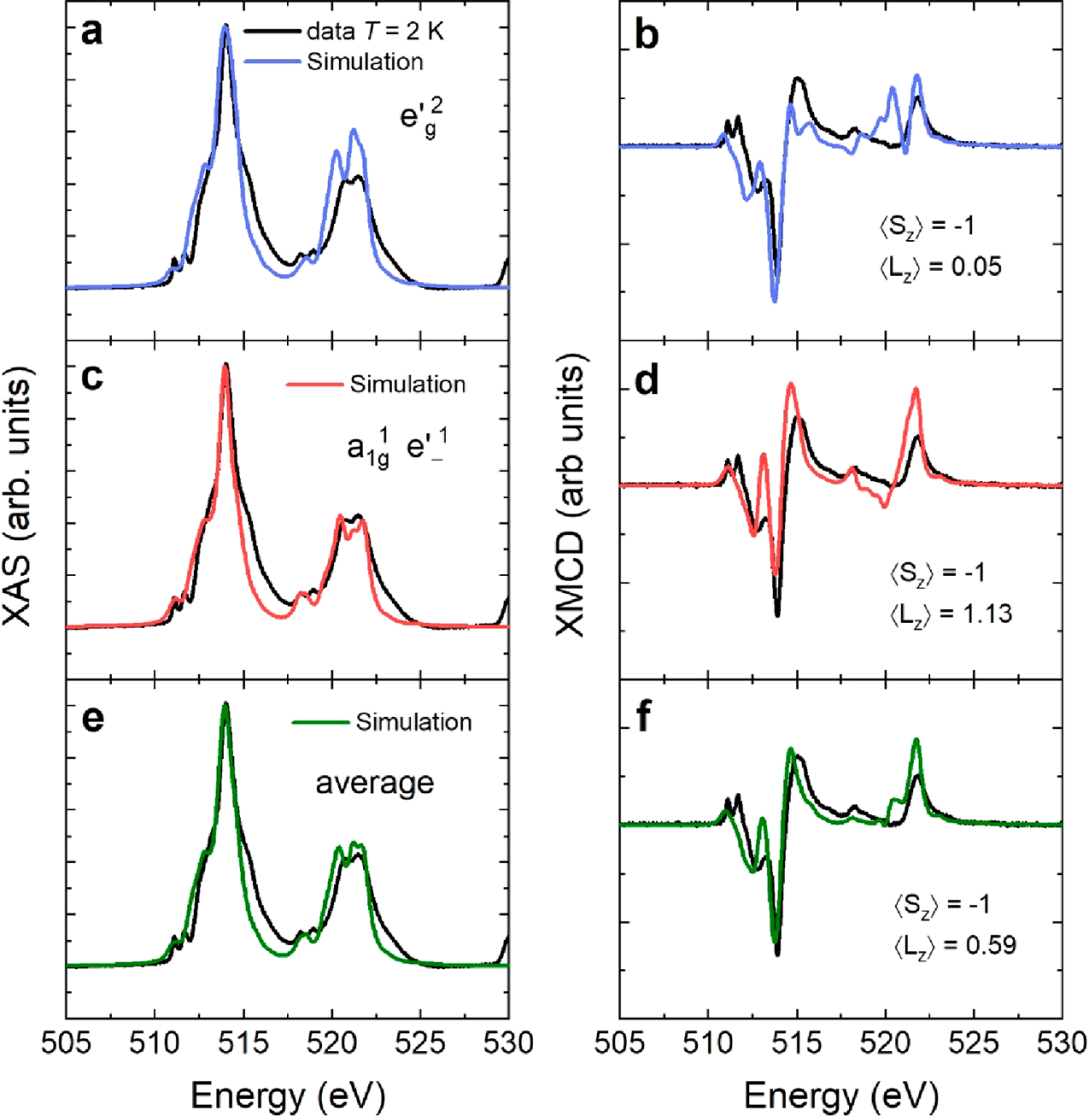
X-ray absorption (left panel) and X-ray magnetic circular dichroism (right panel). The data are plotted in black, while the simulations are in blue, red, and green. (a) and (b) simulations correspond to  eV and a quenched OM. (c) and (d) simulations correspond to  eV and an unquenched OM. (e, f) XAS and XMCD simulation is the average of (a) and (b) simulations which corresponds to an OM of ∼0.6 μ_B_.

Recently an ARPES investigation has revealed a significant occupation of  orbitals, in addition to  orbitals.^34^ In their work, the authors attribute that to V^2+^ at the surface, although their XAS agrees with V^3+^. The argument for this proposition is based on a prediction that  should be fully occupied at the ground state and only the existence of V^2+^ could then explain the observed  occupation. The existence of a ground state with the  orbital occupied and a high OM, as found here following previous works,^17,18,39^ would be a reasonable explanation of the ARPES results.

The importance of many-body effects, solid-state hybridization, and the possible presence of multiple V sites in this system represent a challenging task for the theory. A more accurate spectra description may be achieved fusing advanced configuration interaction techniques or the Bethe–Salpeter equation.^38,41^

In summary, our XMCD results unequivocally demonstrate the existence of an unquenched OM of V in VI_3_, thus resolving the long debate on this issue. Our findings are connected to theoretical models of the V ground state in VI_3_, help us to reveal which orbitals are occupied, and explain the large magnetic anisotropy observed in this system. Using ligand field multiplet simulations, we could show that the ground state of the V ion would agree with inelastic neutron scattering results where two V sites with opposite trigonal distortion were proposed.

## Supporting Information Available

Methods for crystal growth, magnetization measurements, XMCD sum rules calculation, multiplet simulations parameters, XAS/XMCD measurement conditions, and DFT calculations details (PDF)
