# Supplementary material for: Large Orbital Magnetic Moment in VI3
Source: Nano Lett. 2023 Feb 1;23(4):1175–80. doi: 10.1021/acs.nanolett.2c04045 (PMC9951247; doi:10.1021/acs.nanolett.2c04045)
Supplement: Supplementary file 1 — nl2c04045_si_001.pdf [file nl2c04045_si_001.pdf]

Supplementary Information -

Large orbital magnetic moment in VI_3

Dávid Hovančík[1], Jiří Pospíšil[1], Karel Carva[1], Vladimír Sechovský[1], Cinthia Piamonteze[2*]*

[1] Charles University, Faculty of Mathematics and Physics, Department of Condensed Matter Physics, Ke Karlovu 5, 121 16 Prague 2, Czech Republic

[2]Swiss Light Source, Paul Scherrer Institut, CH-5232 Villigen PSI, Switzerland

*Corresponding Author: C. Piamonteze, D. Hovančík

Email: cinthia.piamonteze@psi.ch, hovancik@mag.mff.cuni.cz

METHODS

CRYSTAL GROWTH

The single crystals of VI_3 were prepared by the chemical vapor transport (CVT) method directly from the elements' stoichiometric ratio (1:3). All manipulations with elements as well as the filing of the quartz ampoule were performed in a glove box under Ar inert atmosphere. The degradation process of VI_3 was described in detail by Kratochvilova et al.[1]. The chemical composition of single crystals was verified by scanning electron microscopy (SEM) in combination with an energy-dispersive X-ray detector (EDX).

MAGNETIZATION MEASUREMENTS

We performed detailed magnetization measurements of VI_3 bulk samples at 2 K to check the total value of V moment. The magnetization data was measured in magnetic field up to 7 T using a SQUID magnetometer MPMS7T (*Quantum Design Inc.*). To probe the angular dependence of magnetization in ac^R-plane (at 2 K in 5 T) we used a homemade rotator with rotation axis orthogonal to the applied magnetic field. The angular-depended magnetization was measured in relative units and then scaled to the data measured along the c^R-axis and *ab*-plane. The results are shown in Figure S1. The maximum magnetization value of 1.23 μ_B was found along a direction tilted by ~ 40º from the c^R-axis.

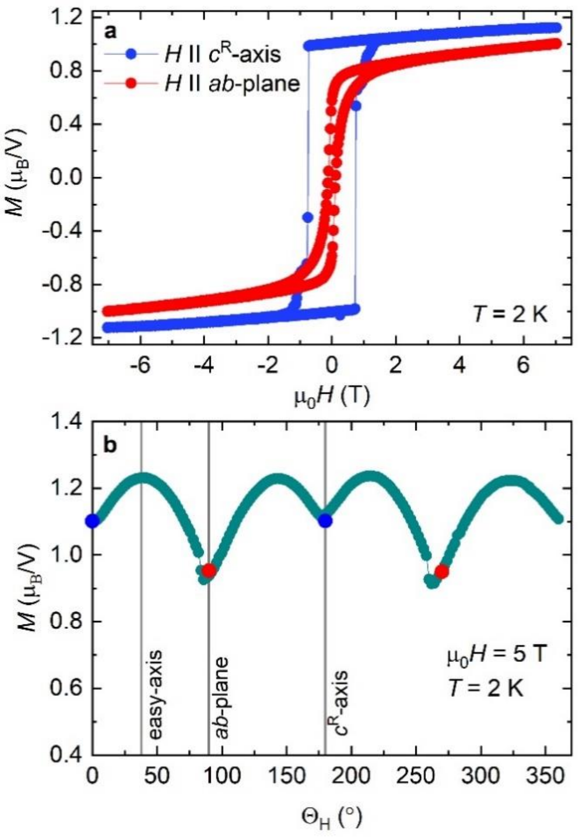

Figure S1 - top: magnetization isotherms measured at 2 K. Bottom: angular dependent magnetization. The angular dependence was scaled according to the absolute values of

magnetization measured along the c^R-axis and *ab*-plane (see blue and red dots). The small discontinuities at around 90° and 270° are of an instrumental origin, most likely caused by the change of magnetic torque.

XMCD sum rules and Oxygen contribution

We used magneto-optical sum rules[2,3] to analyze the direction and value of the projection of an orbital magnetic moment (m_{orb}). The spin sum rule requires separating the XMCD contribution from the L_3 and L_2 edges. Correction factors of the spin sum rule have been calculated for the late transition metals[4,5]. However, for Ti, V, and Cr the overlap is large and no correction factor can be properly calculated. Therefore, we focus only on the orbital sum rules given by the expression below:

$$\widehat{\boldsymbol{P}} \cdot \boldsymbol{m}_{\mathrm{orb}} = -\frac{4}{3}\frac{\mathrm{q}}{\mathrm{r}} n_h \mu_B \qquad (1)$$

where $\widehat{\boldsymbol{P}}$ is the x-ray propagation unit vector, q and r are defined in figure S2, n_h stands for the number of holes per atom in the 3d orbital, μ_B is Bohr magneton. In expression (1) the quantity r is calculated from the XAS defined as the sum of individual spectra measured with left and right circularly polarized X-rays.

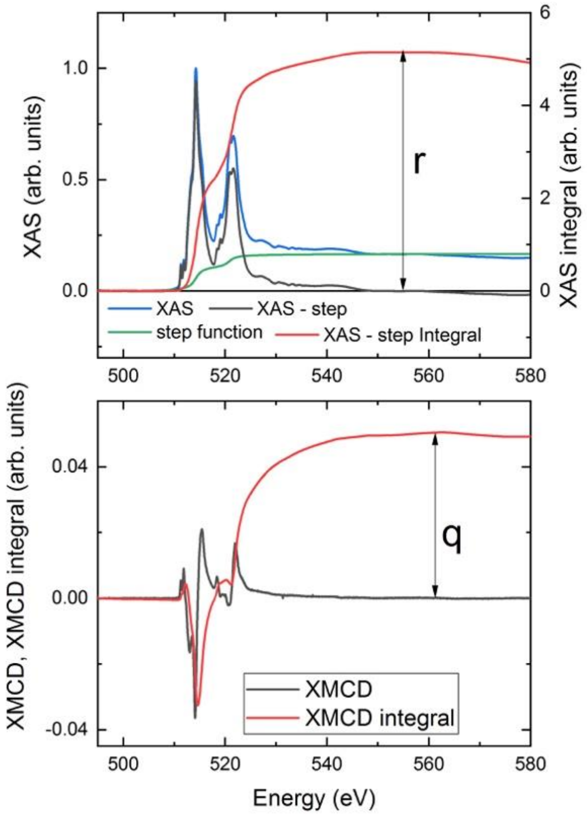

Figure S2- top: XAS and bottom: XMCD measured at 90 K normal incidence, 5 T applied field parallel to the X-ray direction.

Figure S3 shows the XMCD and corresponding integral measured at 2 K, 5 T, normal incidence. The integral value indicated is calculated as the average in the energy region 540-580eV and the standard deviation error bar. This data was used for the OM calculation presented in the manuscript.

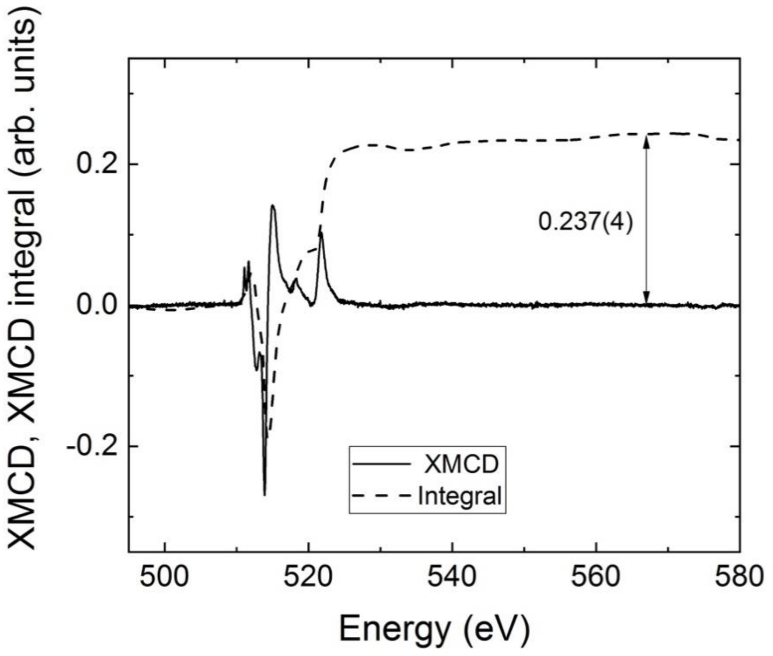

Figure S3 - XMCD measured at 2 K, 5 T normal incidence and corresponding integral. The integral value indicated is the average within the energy range 540-580 eV and the error bar is the standard deviation.

As discussed in the manuscript, the XAS measured at low temperatures presents an additional contribution of the oxygen K-edge. The base pressure of the chamber during the measurements is below 5e-11mbar, so a residual gas containing lots of oxygen is unlikely to be the origin. One of the main differences between the 90 K or 300 K compared to the 2 K measurement is that the TEY drain current is strongly reduced at 2 K, since VI_3 is a semiconductor. The TEY at the V-L_3 absorption is reduced by a factor of almost 40 between 90 K and 2 K. This means that if there is a 2% illumination of the sample plate (which contains oxides) that will not be seen at 90 K since the Vanadium signal is much stronger, but, at 2 K, the Vanadium TEY has approximately the same magnitude as this small contribution from the sample holder. The x-ray beam used is out-of-focus to minimize radiation damage. In this condition, the 4σ vertical footprint of the x-ray beam, which should contain about 95% of the x-ray intensity is roughly

matching the crystal vertical size. Therefore a few % of x-rays illuminating the sample holder is quite plausible.

The O K-edge presence makes a direct calculation of the XAS integral difficult. There are different ways of estimating the XAS integral for the sum rule, which we discuss in sequence. Figure S4 shows the XAS spectra and corresponding integral for the 2 K and 300 K data. One way to calculate the XAS integral is to adjust the step function to the 2 K XAS spectrum around 527 eV, subtracting the step function from the XAS and taking the integral at this energy. The value of the XAS integral is 3.76 and the corresponding OM, if we use the XMCD integral from figure S3 and $n_h = 8$, is 0.67 μ_B. The issue of this method is that the normalization of the step function cannot be done at a high-energy post-edge as it is usually done.

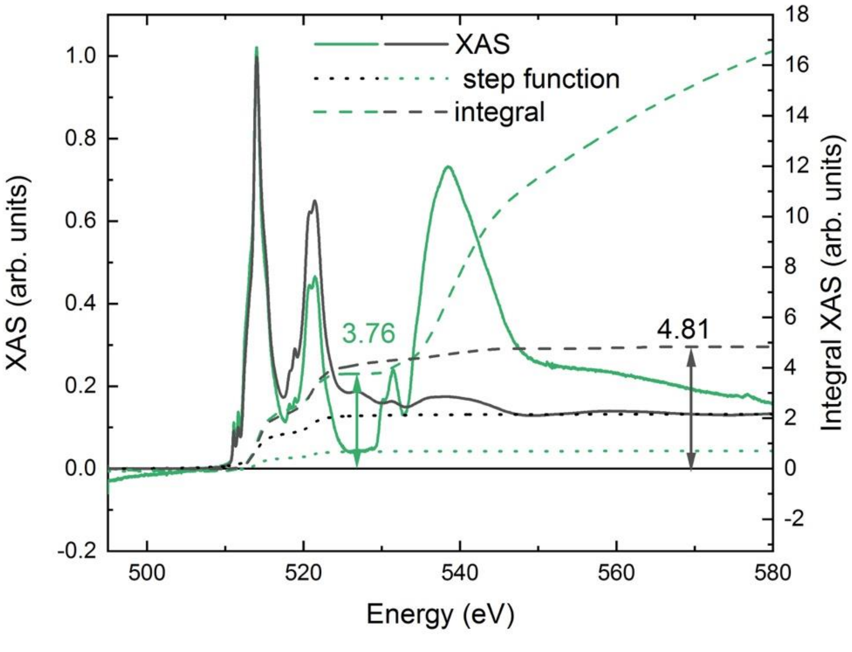

Figure S4 - XAS (continuous line), step function (dotted line) and corresponding integral after step function removal (dashed line) for the spectrum measured at 2 K (green) and 300 K (dark gray) in normal incidence. The values of the respective integrals are given above the arrows at the corresponding energy.

A second method would be to take the integral of the data measured at 300 K, where the oxygen spectrum does not interfere, and the step function can be normalized at high energy. In this case, the step function is normalized around 570eV and the corresponding integral after the step function removal is 4.81. The resulting OM is 0.53 μ_B. This method is better than the previous one because we can calculate the full XAS integral more precisely. The disadvantage is that the XAS at 300 K is not identical to the one at 2 K. As seen in figure S4, the relative ratio of L_3 and L_2 peaks is different.

For this reason, we suggest a third method, which is to estimate the difference in the XAS integral, if the step function is normalized at 527 eV or at 570 eV and use that correction factor to estimate the correct XAS integral for the 2 K spectrum if the step would be normalized at 570 eV. The correction factor was calculated using the 300 K data. As shown in figure S5 the XAS integral of the 300 K data taken at 527 eV, with the step function normalized to the data at this energy, is 3.53. Therefore, the integral taken just above the L_2 edge corresponds to 3.53/4.81*100=73% of the integral taken at 570 eV. Using this relative difference between the 300 K integrals at two different energy points, we can attempt to correct the 2 K XAS integral, which can only be taken in a restricted energy range. We obtain: 3.76/0.73 = 5.12 as an estimate for the 2 K XAS integral value if there would be no Oxygen contribution to the spectrum. If we use this value for the XAS integral, we obtain an OM of 0.49 μ_B.

In summary, we present three different methods to calculate the XAS integral and the OM. Each method has its advantages and disadvantages. The main message of these calculations is to show that the OM for vanadium in VI_3 is significantly large no matter which method we use. As a representative estimate of OM, we took the average value of the results of the three aforementioned methods and used the standard deviation among them as the error bar. This comes down to OM of **0.6(1) μ_B.**

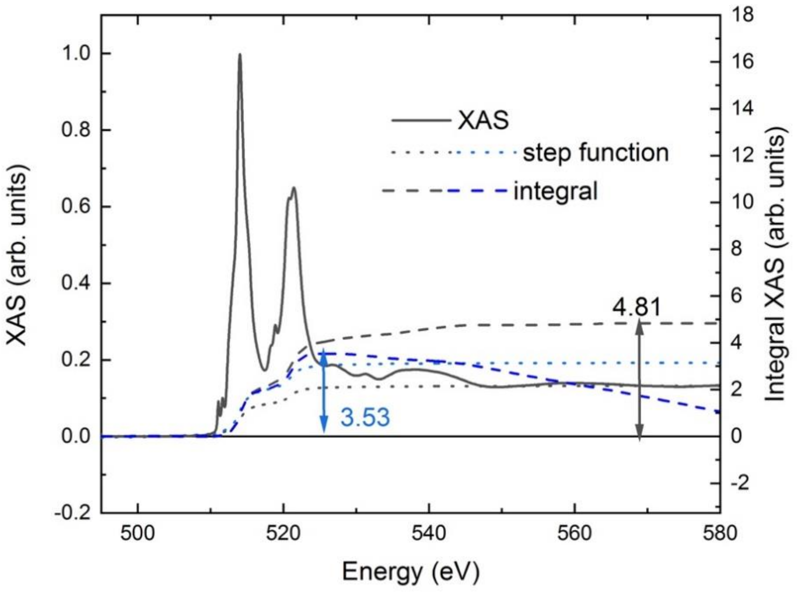

Figure S5 - XAS measured at RT normal incidence (continuous line). The dotted line shows two different options for the step function: aligning with the XAS at 527 eV or at 570 eV. The dashed lines represent the corresponding integral, following the color of the step function.

MULTIPLET SIMULATIONS

The X-ray absorption spectra and x-ray magnetic circular dichroism spectra were simulated for V^{3+} d^2 using ligand field multiplet simulations based on atomic Hartree-Fock calculations using Cowan's code[6] followed by chain symmetry proposed by Butler[7]. This approach has been used for the first time by Thole and van der Laan for the description of x-ray absorption spectra[8]. The local V symmetry used in the simulations was D_{3d} which is reduced to C_3 when the magnetic moment is added. Slater Integrals were reduced by 50% from Hartree-Fock values. All parameters used are summarized in Table S1. The *d*-SOC was set to 13 meV following Lane *et al.*[9], which corresponds to 50% of the atomic value. The *p*-SOC was increased by 6% to fit with the experimental splitting of the L_3 and L_2 edges. In the main text, we refer to the size of trigonal distortion using the energy splitting of the t_{2g} levels

$$E(e_g') - E(a_{1g}) = 3D_\sigma + 20/3\, D_\tau = D_{trg} \quad (2)$$

$F^2(d,d)$ / $F^4(d,d)$ ground state (eV)	5.06 / 3.18
$F^2(d,d)$ / $F^4(d,d)$ final state (eV)	5.49 / 3.44
$F^2(p,d)$ / $G^1(p,d)$ / $G^3(p,d)$ final state (eV)	3.03 / 2.20 / 1.25
$10D_q$ (eV)	1.5
D_σ fig 4a,b / fig 4c,d (eV)	-0.05 / 0.10
D_τ (eV)	0.0
d-SOC ground state / final state (eV)	0.013 / 0.018
p-SOC (eV)	4.928

Table S1 - parameter used in multiplet simulations shown in Figure 4 of main text.

The energy levels in trigonal symmetry are related to the CFS parameters as shown in equations S(3-5).

$$E(e') = -4 \times D_q + D_\sigma + {}^{2}/_{3} \times D_\tau \quad (3)$$

$$E(a_{1g}) = -4 \times D_q - 2 \times D_\sigma - 6 \times D_\tau \quad (4)$$

$$E(e_g) = 6 \times D_q + {}^{7}/_{3} \times D_\tau \quad (5)$$

The choice of CFS parameters was based on DFT calculations and previous results, as from Lane et al.[9]

XAS, XMCD MEASUREMENTS

X-ray magnetic circular dichroism measurements were carried out using the EPFL-PSI end-station in the X-Treme beamline[10], Swiss Light Source. The crystals were mounted inside a

glove box and cleaved for a fresh surface. Then the crystals were transferred in an inert atmosphere from the glove box to the load lock, where they were cleaved for a second time and then pumped down. To improve conductivity for the total electron yield measurements, the crystals were capped with a few nm carbon layer evaporated *in situ* in the ultra-high vacuum sample preparation chamber. The measurements shown were done with the x-rays perpendicular to the exfoliation plane (parallel to the *c*-axis in rhombohedral structure, c^R).

Figure S6 shows two measurements done during the same beamtime with a time difference of 6.5 hours. These measurements show that no sign of change in the XAS nor XMCD is observed due to the long incidence time of the x-rays.

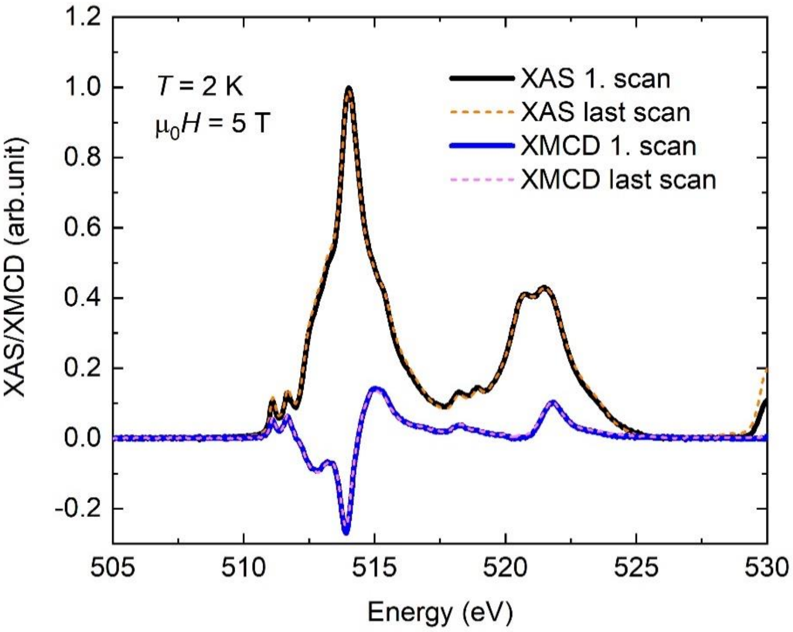

Figure S6 - XAS and XMCD measurements done in the same crystal 6.5 hrs. apart in time showing no sign of radiation damage.

DFT CALCULATIONS

Density functional theory (DFT) calculations employed the full-potential linear augmented plane wave (FP-LAPW) method, as implemented in the band structure program ELK[11]. The

generalized gradient approximation (GGA) parametrized by Perdew-Burke- Ernzerhof[12] has been used to determine the exchange-correlation potential. SOC is known to play a key role in the formation of OM and has been included in the calculation. DFT simulations utilizing GGA-PBE have already successfully described quasi-2D compound VI_3[13]. The full Brillouin zone has been sampled by 10 × 10 × 5 k-points and the convergence w.r.t. k-mesh density has been verified. An increased accuracy of expansion into spherical harmonics has been used with l_{max}=14. The simulation was performed for a bulk system consisting of weakly coupled layers. Since the material is known to be a Mott insulator, we have included the effect of electron-electron correlations in terms of the Hubbard correction term U = 4.3 eV [14,15] together with Hund's rule exchange parameter J = 0.8 eV, acting on 3*d* electrons of V. Double counting was treated in the fully localized limit.

For the expansion into spherical harmonics basis the quantization of orbital momentum eigenstates is chosen along the 3-fold axis C_3, where also the external magnetic field points (not the C_4 axis as commonly used for quantization in O_h). Then the a_{1g} state has a particularly simple form in the basis of complex spherical harmonics (quantum number l = 2 omitted further):

$$|a_{1g}\rangle = |m=0\rangle^{16} \tag{6}$$

The states $|e'_+\rangle$, $|e'_-\rangle$ originate from the following linear combinations of spherical harmonics, the former t_{2g} states of the cubic system[17]:

$$|e'_+\rangle = \sqrt{\frac{2}{3}}|m=2\rangle - \sqrt{\frac{1}{3}}|m=-1\rangle \tag{7}$$

$$|e'_-\rangle = \sqrt{\frac{2}{3}}|m=-2\rangle + \sqrt{\frac{1}{3}}|m=1\rangle \tag{8}$$

These are eigenstates of L_z operator with values ±1, and therefore remain degenerate w.r.t. trigonal distortion, but are split by SOI.

NOTE S1

For temperature-induced effects energy 5.6 meV (~ 65 K) is high enough to be not excited at considered temperatures (2 K). However, even a small distortion of the lattice introduces significantly larger changes in total energy. Therefore, in the presence of various point defects or stacking faults, the state previously located 5.6 meV higher could become favorable. Another aspect is that in this situation we have a local minimum separated by 5.6 meV from the global minimum. These appear to be relatively far from each other in phase space, and there is also the metallic solution as another local minimum. This increases the risk that self-consistent calculation finds local minimum that is not a true global minimum, which could explain why different results were got by different groups.

1 Kratochvílová, M. *et al.* The surface degradation and its impact on the magnetic properties of bulk VI3. *Materials Chemistry and Physics* **278**, 125590, doi:10.1016/j.matchemphys.2021.125590 (2022).

2 Thole, B. T., Carra, P., Sette, F. & van der Laan, G. X-ray circular dichroism as a probe of orbital magnetization. *Physical Review Letters* **68**, 1943-1946, doi:10.1103/PhysRevLett.68.1943 (1992).

3 Carra, P., Thole, B. T., Altarelli, M. & Wang, X. X-ray circular dichroism and local magnetic fields. *Physical Review Letters* **70**, 694-697, doi:10.1103/PhysRevLett.70.694 (1993).

4 Teramura, Y., Tanaka, A. & Jo, T. Effect of Coulomb Interaction on the X-Ray Magnetic Circular Dichroism Spin Sum Rule in 3 d Transition Elements. *Journal of the Physical Society of Japan* **65**, 1053-1055, doi:10.1143/JPSJ.65.1053 (1996).

5 Piamonteze, C., Miedema, P. & de Groot, F. M. F. Accuracy of the spin sum rule in XMCD for the transition-metal *L* edges from manganese to copper. *Physical Review B* **80**, 184410, doi:10.1103/PhysRevB.80.184410 (2009).

6 Cowan, R. D. *The Theory of Atomic Structure and Spectra*. (University of California Press, 1981).

7 STEGER, E. Philip H. Butler. Point group symmetry applications. Methods and tables. plenum press New York and London, 1981 567 seiten, Preis: US $ 55.00. *Crystal Research and Technology* **18**, 404-404, doi:https://doi.org/10.1002/crat.2170180316 (1983).

8 van der Laan, G. & Thole, B. T. Strong magnetic x-ray dichroism in 2p absorption spectra of 3d transition-metal ions. *Physical Review B* **43**, 13401-13411, doi:10.1103/PhysRevB.43.13401 (1991).

9 Lane, H. *et al.* Two-dimensional ferromagnetic spin-orbital excitations in honeycomb VI_3. *Physical Review B* **104**, L020411, doi:10.1103/PhysRevB.104.L020411 (2021).

10 Piamonteze, C. *et al.* X-Treme beamline at SLS: X-ray magnetic circular and linear dichroism at high field and low temperature. *Journal of Synchrotron Radiation* **19**, 661-674, doi:doi:10.1107/S0909049512027847 (2012).
11 *The Elk Code, https://elk.sourceforge.io/*, <url: http://elk.sourceforge.net/.> (
12 Perdew, J. P., Burke, K. & Ernzerhof, M. Generalized Gradient Approximation Made Simple *Physical Review Letters* **78**, 1396-1396, doi:10.1103/PhysRevLett.78.1396 (1997).
13 Sandratskii, L. M. & Carva, K. Interplay of spin magnetism, orbital magnetism, and atomic structure in layered van der Waals ferromagnet **VI_3**. *Physical Review B* **103**, 214451, doi:10.1103/PhysRevB.103.214451 (2021).
14 Anisimov, V. I., Zaanen, J. & Andersen, O. K. Band theory and Mott insulators: Hubbard U instead of Stoner I. *Physical Review B* **44**, 943-954, doi:10.1103/PhysRevB.44.943 (1991).
15 Anisimov, V. I., Aryasetiawan, F. & Lichtenstein, A. I. REVIEW ARTICLE: First-principles calculations of the electronic structure and spectra of strongly correlated systems: the LDA+ U method. *Journal of Physics Condensed Matter* **9**, 767-808, doi:10.1088/0953-8984/9/4/002 (1997).
16 Khomskii, D. I. *Transition Metal Compounds*. (Cambridge University Press, 2014).
17 Ballhausen, C. J. *Introduction to Ligand Field Theory*. McGraw-Hill, New York, (1962).
